# Stepwise Implementation of 2D Synthesized Screening Mammography and Its Effect on Stereotactic Biopsy of Microcalcifications

**DOI:** 10.3390/diagnostics13132232

**Published:** 2023-06-30

**Authors:** Karen E. Gerlach, Kanchan Ashok Phalak, Ethan O. Cohen, Kiran N. Chang, Roland Bassett, Gary J. Whitman

**Affiliations:** 1 Department of Breast Imaging, MD Anderson Cancer Center, 1155 Pressler St. Unit 1350, Houston, TX 77030, USA; 2 Department of Radiology, University of Texas Health Science Center, 6431 Fannin St, Houston, TX 77030, USA; 3 Biostatistics Department, MD Anderson Cancer Center, 1515 Holcombe Blvd., Houston, TX 77030, USA

**Keywords:** breast cancer, synthesized mammography, stereotactic biopsy, microcalcifications, screening mammography

## Abstract

Rationale and Objectives: Information evaluating the efficacy of 2D synthesized mammography (2Ds) reconstructions in microcalcification detection is limited. This study used stereotactic biopsy data for microcalcifications to evaluate the stepwise implementation of 2Ds in screening mammography. The study aim was to identify whether 2Ds + digital breast tomosynthesis (DBT) is non-inferior to 2D digital mammography (2DM) + 2Ds + DBT, 2DM + DBT, and 2DM in identifying microcalcifications undergoing further diagnostic imaging and stereotactic biopsy. Materials and Methods: Retrospective stereotactic biopsy data were extracted following 151,736 screening mammograms of healthy women (average age, 56.3 years; range, 30–89 years), performed between 2012 and 2019. The stereotactic biopsy data were separated into 2DM, 2DM + DBT, 2DM + 2Ds + DBT, and 2Ds + DBT arms and examined using Fisher’s exact test to compare the detection rates of all cancers, invasive cancers, DCIS, and ADH between modalities for patients undergoing stereotactic biopsy of microcalcifications. Results: No statistical significance in cancer detection was seen for 2Ds + DBT among those calcifications that underwent stereotactic biopsy when comparing the 2Ds + DBT to 2DM, 2DM + DBT, and 2DM + 2Ds + DBT imaging combinations. Conclusion: These data suggest that 2Ds + DBT is non-inferior to 2DM + DBT in detecting microcalcifications that will undergo stereotactic biopsy.

## 1. Introduction

Digital breast tomosynthesis (DBT) detects more breast cancers than 2D digital mammography (2DM) [1,2]. However, 2DM remains more sensitive than DBT alone for calcifications, and 2DM and DBT have historically been performed together [3,4]. In practice, when indeterminate calcifications are detected on mammography, the patient is called back for additional views using 2D magnification for additional characterization. Synthetic 2D mammogram (2Ds) algorithms were designed to replace the need for additional 2DM during screening mammography. DBT images are reconstructed into 2Ds images. The use of 2Ds eliminates the need to perform 2DM, which decreases the effective glandular radiation dose and the imaging time when compared to 2DM + DBT [5,6].

## 2. Prior 2Ds Research

Prior studies have shown that the performance of 2Ds appears to be comparable to that of 2DM; however, most studies have focused on non-calcified lesions [1,7,8,9,10,11]. The performance of 2Ds with microcalcifications is less clear. Zuley et al. [11] analyzed 123 patients and suggested that 2Ds and 2DM were comparable in terms of performance. However, only 19 calcification cases were included in the study by Zuley et al. [11]. Zuckerman et al. [9] assessed 2Ds versus 2DM + DBT regarding performance among 20,927 screening mammograms, with 310 recalls for calcifications, and showed fewer call-backs for 2Ds + DBT versus 2DM + DBT. Although the DCIS detection rate between the two groups was not significantly different, the authors raised concerns that 2Ds may overlook calcifications [9]. Peters et al. [12] assessed radiologist performance via in vitro modeling and concluded that 2DM was superior to 2Ds, questioning whether 2Ds could replace 2DM. Another study assessed 72 consecutive screening mammograms recalled for calcifications over 14 months and found that 2Ds + DBT and 2DM performed similarly in the detection of microcalcifications at screening mammography [13]. Similar results were obtained in another reader study using 160 subjects [14]. Furthermore, a meta-analysis of 2Ds versus 2DM suggests comparable accuracy between modalities, although the meta-analysis may have limitations due to the heterogeneous study designs [10]. To date, no large study has compared 2DM, 2DM + DBT, 2DM + 2Ds + DBT, and 2Ds + DBT performance with respect to the identification of microcalcifications. 

## 3. Research Challenges

Challenges in interpreting 2Ds studies include different implementation approaches and algorithms which might contribute to study variances [8,15]. There are reports of decreased 2Ds call-back rates attributed to decreased calcification visibility [16]. Additionally, obtaining satisfactory volumes of cases to study 2Ds performance for microcalcifications alone may be challenging [15].

## 4. Changing Modalities

Learning curves are expected with new technologies and, therefore, some facilities obtain 2DM and 2Ds to facilitate prior imaging comparisons [15]. Changing modalities can affect mammographic interpretations, as seen when transitioning from film screen mammography to 2DM [17]. We used a stepwise transition when implementing 2Ds at a large, community-based hospital, utilizing 2DM + DBT, 2DM + 2Ds + DBT, and then 2Ds + DBT. The stepwise transition of 2Ds incorporation was intended to support radiologist learning and facilitate prior imaging comparisons. Throughout the study, 2DM was also performed, based on patient preference. 

The transition from 2DM + DBT to 2DM + 2Ds + DBT to 2Ds + DBT was examined for microcalcifications undergoing stereotactic biopsy. Our hypothesis was that stepwise transition to 2Ds would show non-inferiority in detecting microcalcifications that would undergo stereotactic biopsy.

## 5. Methods

Institutional Review Board approval was obtained per Health Insurance Portability and Accountability Act guidelines. Informed consent was waived by the Institutional Review Board.

### 5.1. Design and Patient Selection

This study is a retrospective review of data extracted from 151,736 screening mammograms of healthy women (average age, 56.3 years; range, 30–89 years). Imaging was performed at a single facility on the same imaging system (Selenia Dimensions 2D/3D System; Hologic, Marlborough, MA, USA). Units were capable of performing both 2DM and DBT. Synthesized reconstructions were performed using software module version 1.8.2 (Hologic, Marlborough, MA, USA).

Patients self-selected to receive 2DM or DBT throughout the entire study period. Patients selecting DBT were made aware of a possible charge if DBT was not covered by their insurance carriers, which may have affected their decisions. The 2Ds imaging was implemented in a stepwise fashion. The radiologist training was largely self-directed. The 2DM + DBT views were obtained from 1 March 2012 through 19 March 2015. The 2DM + 2Ds + DBT modality was implemented on 20 March 2015. Only the 2Ds + DBT modality was performed after 8 April 2017. Following recall from screening for calcifications, all patients underwent magnification mammography with 2DM in the craniocaudal and the lateromedial or mediolateral views.

### 5.2. Data Selection

The women in the study population underwent 151,736 screening mammograms over seven years (1 March 2012–28 February 2019). The stereotactic biopsy data were extracted, and the screening mammography modality was determined for each subject. The study groups are as follows: 2DM + DBT (three years), 2DM + 2Ds+ DBT (two years), and 2Ds + DBT (two years). Patients who selected 2DM only were grouped into the same three time periods.

The patients included in this study were females, with no prior cancer history, who underwent stereotactic biopsy of microcalcifications without associated masses during the specified time periods. The corresponding medical records, imaging reports, and images were reviewed. Stereotactic biopsies were performed at a single facility on the same biopsy system (Selenia Dimensions 2D/3D System; Hologic, Marlborough, MA, USA). Vacuum-assisted 9-gauge cores were obtained using an Eviva needle (Hologic, Marlborough, MA, USA). The patients excluded from this study were those with screening mammograms performed at another institutional site or at a different institution, those with biopsies performed at other institutions, those lost to follow-up, and those who had a remote or recent diagnosis of breast malignancy.

There were 28 interpreting radiologists averaging 15.4 years in practice (mean, 13 years; range, 4–32 years). All of the radiologists were dedicated breast radiologists.

The patients were monitored for interval cancers for two years except for the final year, for which 1.6 years of follow-up data were available. The primary outcome was to assess the stereotactic biopsy results for calcifications with each modality.

### 5.3. Statistics

The data were examined using Fisher’s exact test to compare the detection rates of all cancers, invasive cancers, DCIS, and ADH between modalities for patients undergoing stereotactic biopsy of microcalcifications (*n* = 832). R version 3.6.1 (The R Foundation) was used for all statistical analysis, and a significance level of 0.05 was used. Multiple biopsy locations were treated as individual biopsy sites in the analysis. 

## 6. Results

**Figure 1:**
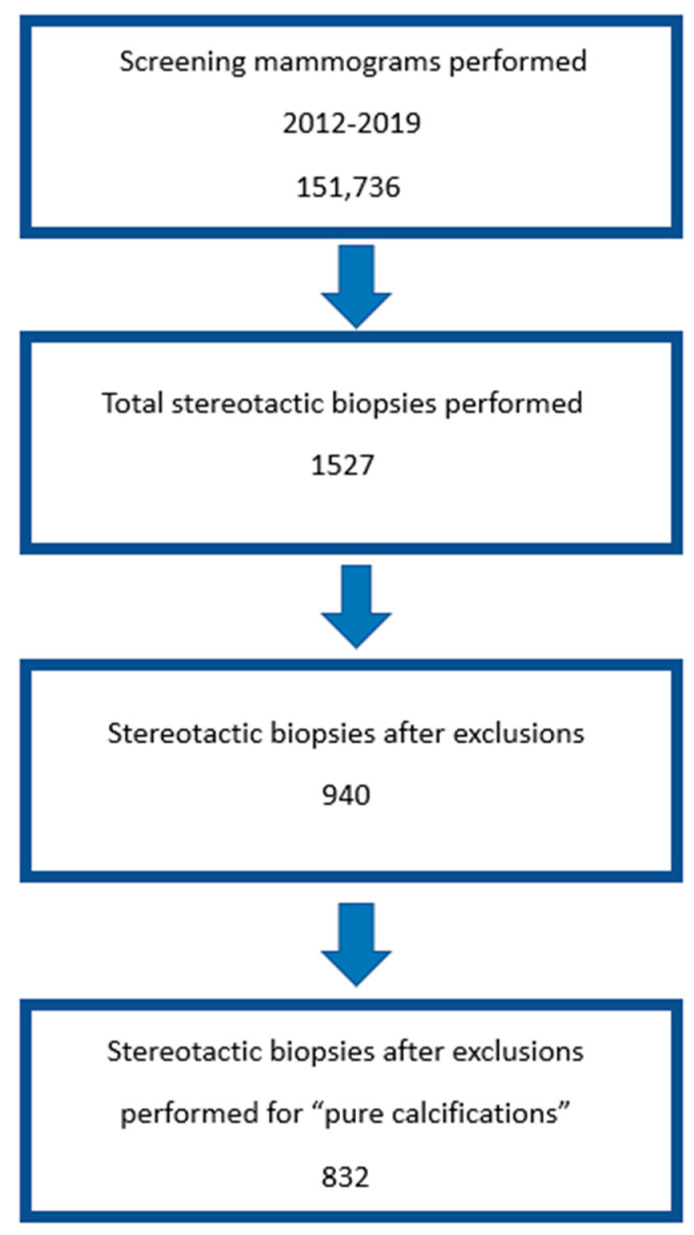
Schema of the experimental design. A patient pool of 151,736 screening mammograms from 2012–2019 was used. The stereotactic biopsy data for 1527 biopsies were extracted. In all, 940 patients remained after the experimental exclusion criteria were applied (587 patients were excluded for screenings performed at other locations, personal history of breast cancer, or incomplete information). Of these, 832 patients met the criteria for microcalcifications (108 were excluded due to masses, asymmetries, or architectural distortions).

**Figure 2:**
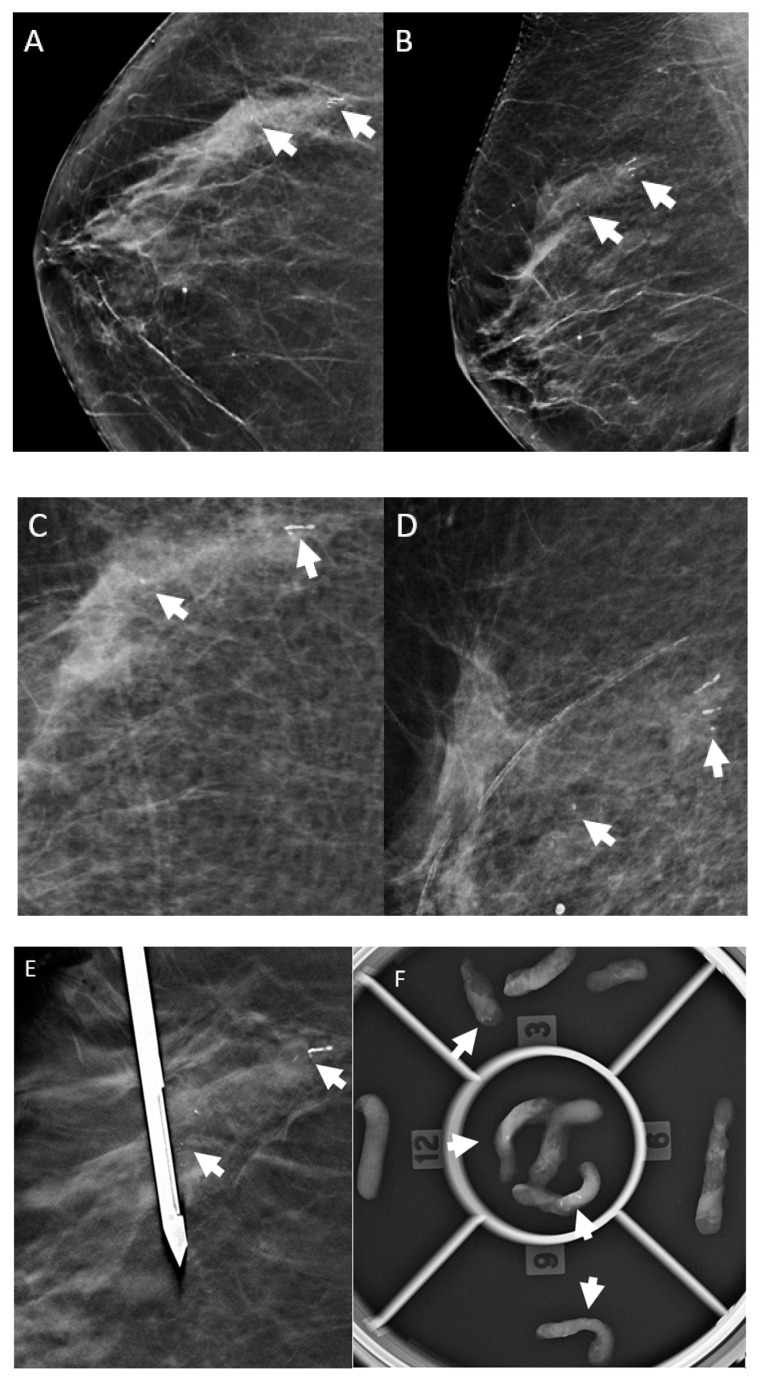
Screening mammography in a 73-year-old woman utilizing 2D synthetic views: (**A**) right craniocaudal view and (**B**) right mediolateral oblique view. The arrows indicate new linear calcifications. The 2D magnification views further characterize the calcifications seen on the screening mammography images in (**A**,**B**). (**C**) Magnification craniocaudal view. (**D**) Magnification lateromedial view. The arrows indicate new calcifications. (**E**) Post-fire stereotactic biopsy image. The vacuum-assisted, stereotactic biopsy device was positioned in the region of the targeted calcifications (arrows). (**F**) The specimen radiograph shows cores containing calcifications (arrows). Pathology showed grade 3 estrogen-receptor-positive, progesterone-receptor-positive ductal carcinoma in situ associated with the calcifications.

The data were extracted from a pool of 151,736 screening mammograms over seven years (Figure 1). These screening mammograms resulted in 1527 stereotactic biopsies. The total number of stereotactic biopsies per year ranged from 151 to 285 (average = 218). Of these stereotactic biopsies, 940 biopsies met the inclusion criteria, and of these, 832 biopsies were performed for microcalcifications without associated masses (Figure 2). In all, 428 stereotactic biopsies were performed in the 2DM arm (Table 1 and Table 2), and 404 stereotactic biopsies were performed in the DBT arms (Table 3). Masses, asymmetries, and architectural distortions were excluded from the analysis. The study time periods were distinguished by the tomosynthesis modality used (2DM + DBT, 2DM + 2Ds + DBT, or 2Ds + DBT). Each category comprised three, two, and two years of data, respectively. The first time period (three years) was accounted for in the proportion analysis. Each modality group contained 207 to 380 patients per time period (Table 2a–c). Comparison to the 2DM group was performed for each time period.

### 6.1. Modality Comparison for Microcalcifications

The only statistically significant value in the modality comparison was ADH in 2012–2015, where *p* = 0.020 (Table 1). More patients were diagnosed with ADH with 2DM (*n* = 23) than with 2DM + DBT (*n* = 7). The remaining groups, 2015–2017 (2DM + 2Ds + DBT) and 2017–2019 (2Ds + DBT), were not statistically significantly different (Table 1). More invasive cancers were diagnosed in 2017–2019 with 2Ds + DBT (*n* = 7) versus 2DM (*n* = 1) (*p* = 0.263), but this was not statistically significant (Table 1).

### 6.2. Lesion Comparison by Year for Calcifications on 2DM

Statistical analysis was performed over the three time periods, which corresponded to the different modality combinations in use: 2012–2015, 2015–2017, and 2017–2019 (Table 2). Here, 2DM was assessed over each time period for the diagnosis of all cancers, invasive cancers, DCIS, and ADH. There were no statistically significant differences in the 2DM group over time (Table 2).

### 6.3. Lesion Comparison by Year for Calcifications with Tomosynthesis Screening

The lesion comparison by year was repeated for all tomosynthesis modalities (Table 3). The diagnoses of all cancer, invasive cancer, DCIS, and ADH lesions were not statistically significantly different (Table 3). The lack of statistically significant differences in the calcification group suggests that 2Ds + DBT is non-inferior to 2DM + DBT for the identification of microcalcifications undergoing stereotactic biopsy.

The patient characteristics were compared by year and modality. For the continuous parameters, there was no evidence of a significant difference in the age of the patients across each time period and modality category used (Table 4). For the categorical parameters, there was strong evidence of an association among years/modalities among women using hormones and possessing prior mammograms (Table 5). Patients in 2015 and 2017 with 2D imaging alone were more likely to have received hormone therapy. Patients in 2012 with 2D alone and patients in 2017 with 2D alone were more likely to have had prior mammograms.

For continuous parameters, for each year and modality, the table presents the number of patients (“N”), the minimum (“Min”) and maximum (“Max”) values, the quartiles (“q1” and “q3”), the median (“Med.”), the mean (“Mean”), and the standard deviation (“SD”), along with the number of missing values (“NA”), if any. For categorical parameters, the table presents for each year/modality the number of patients in each category/level.

For the categorical parameters, there is strong evidence of an association among years/modalities and both hormones and prior mammograms. Patients in 2015 and 2017 with 2D imaging alone were more likely to have received hormone therapy. Patients in 2012 with 2D alone and patients in 2017 with 2D alone were more likely to have had prior mammograms.

## 7. Discussion

This study extracted data from a screening mammography population of 151,736 women to obtain a population of patients with microcalcifications. This study used stereotactic biopsy data for microcalcifications to evaluate the stepwise practice implementation of 2Ds.

## 8. Assessment of 2DM Data

The assessment of 2DM data was used throughout all study time periods and confirmed a stable comparison population over time with a stable cancer detection rate. In our study, there was likely patient self-selection of 2DM versus DBT due to variable insurance coverage. The proportion of patients who selected DBT increased over the study period, and there were several possible causes. In this community setting, between March 2012 and February 2018, patients opting for DBT were notified that their insurance companies might not cover DBT and that the women may be required to pay a surcharge. In 2017, the Texas State Legislature mandated that health insurance companies must cover screening DBT starting on 1 January 2018. These economic factors may have accounted for the increasing trend of DBT over 2DM during the study. Other reasons include increased patient understanding of DBT’s benefits in cancer detection and patients declining DBT due to radiation concerns.

The statistical significance seen in the modality comparison for the 2012–2015 2DM arm for increased ADH diagnosis may represent the relatively low numbers of women called back for calcifications that required stereotactic biopsy. In total, 151,736 screening mammograms were performed, of which data for 940 stereotactic biopsies were utilized after exclusions. This study also assessed clinically significant calcifications. Those calcifications classified as benign were not included. This may represent opportunities for further study.

## 9. 2Ds Non-Inferiority

To address the primary endpoint, our data suggest that 2Ds + DBT is non-inferior to 2DM + DBT and 2DM + 2Ds + DBT in identifying calcifications without masses that underwent stereotactic biopsy. These findings are noted by the lack of statistical significance in the stereotactic data among DBT modalities (Table 3). Thus, 2Ds reconstructions are non-inferior to 2DM when evaluating calcifications. This was reflected among all calcification lesions including total cancers, invasive cancers, DCIS, and ADH. These data also reveal that stepwise implementation does not appear to improve 2Ds performance. This stepwise transition may be unnecessary for identifying calcifications undergoing stereotactic biopsy.

## 10. Follow-Up

We reviewed patient follow-up data for up to two years to detect any interval cancers. Two additional new, unrelated ipsilateral cancers were diagnosed from the 2017–2019 group. One patient had screening mammography with 2DM and the other had screening mammography with 2Ds + DBT.

## 11. Limitations

This study was not designed to evaluate the recall rates or the false negative rates from screening mammography. Patients in whom recalled calcifications demonstrated classic benign characteristics and were then returned to screening mammography were not included in this study. Similarly, lesions that were better suited for biopsy under sonographic guidance were also excluded from this study. Another limitation was that following recall for calcifications, all patients underwent 2DM magnification mammography in the craniocaudal and the lateromedial or the mediolateral views. This study did not have a comparison for a non-stepwise approach. Furthermore, this is a single-vendor study, which can limit the generalizability of the results. Future, larger, multi-center studies may be performed to increase the statistical power.

## 12. Advantages of 2Ds

The primary advantage of the 2Ds technique is reduction in the radiation dose. During implementation of 2Ds, radiologists must become comfortable in assessing whether calcifications are true calcifications or artifacts on the synthesized images. Our results suggest that a stepwise 2Ds implementation approach does not add any benefit to the detection of calcifications that require stereotactic biopsy. However, radiologists might gain greater psychological comfort using a stepwise implementation approach. Due to the decreased frequency of microcalcification lesions relative to masses and asymmetries, the benefits of directly implementing 2Ds technology outweigh the additional, small, radiation risk of continuing concurrent 2DM. While the radiation risk of 2DM + DBT is still within the acceptable range, the benefits of 2Ds may outweigh the risks of additional radiation exposure from a population-based standpoint.

## 13. Conclusions

Direct implementation of 2Ds technology can be performed and is non-inferior to 2DM in the detection of calcifications that subsequently undergo stereotactic biopsy.

## Figures and Tables

**Table 1:** Modality comparison for microcalcification by pathology. Lesion detection rates by modality type for patients with microcalcifications (*n* = 832) undergoing stereotactic breast biopsy. Data compare the rates of lesion detection between modalities used during each time period: a. 2DM vs. 2DM + DBT, b. 2DM vs. 2DM + 2Ds + DBT, or c. 2DM vs. 2Ds + DBT.

		All Cancer		Invasive Cancer		DCIS		ADH	
**a. 2012–2015**									
Modality	Total	No	Yes	No	Yes	No	Yes	No	Yes
2D FFDM	210	161	49	203	7	168	42	187	23
2D FFDM + 3DT	170	133	37	165	5	138	32	163	7
Total	380	294	86	368	12	306	74	350	30
*p* =		0.805		1.000		0.796		0.020	
**b. 2015–2017**									
Modality	Total	No	Yes	No	Yes	No	Yes	No	Yes
2D FFDM	142	115	27	137	5	120	22	132	10
2D FFDM + s2D + 3DT	103	85	18	101	2	87	16	92	11
Total	245	200	45	238	7	207	38	224	21
*p* =		0.868		0.702		1.000		0.359	
**c. 2017–2019**									
Modality	Total	No	Yes	No	Yes	No	Yes	No	Yes
2D	76	58	18	75	1	59	17	66	10
s2D + 3DT	131	95	36	124	7	102	29	121	10
Total	207	153	54	199	8	161	46	187	20
*p* =		0.624		0.263		1.000		0.226	

Key: 2DM, 2D digital mammography; 2Ds, synthesized 2D mammography; DBT, digital breast tomosynthesis; DCIS, ductal carcinoma in situ; ADH, atypical ductal hyperplasia. Positive pathology results for each category are indicated by Yes. Negative pathology results for each category are indicated by No.

**Table 2:** Lesion comparison by year for microcalcification lesions detected via 2DM. Summary of lesion detection rates for patients with microcalcifications (*n* = 428) on 2DM screening who underwent stereotactic biopsy. The *p* values compare the detection rates between years.

		All Cancers		Invasive Cancers		DCIS		ADH	
Years	Total	No	Yes	No	Yes	No	Yes	No	Yes
2012–2015	210	161	49	203	7	168	42	187	23
2015–2017	142	115	27	137	5	120	22	132	10
2017–2019	76	58	18	75	1	59	17	66	10
Total	428	334	94	415	13	347	81	385	43
*p* =		0.595		0.807		0.392		0.280	

Key: 2DM, 2D digital mammography; DCIS, ductal carcinoma in situ; ADH, atypical ductal hyperplasia. Positive pathology results for each category are indicated by Yes. Negative pathology results for each category are indicated by No.

**Table 3:** Lesion comparison by year for microcalcifications detected via digital breast tomosynthesis screening. This summarizes lesion detection rates for patients with microcalcifications (*n* = 404) undergoing stereotactic biopsy and tomosynthesis screening (2DM + DBT, 2DM + 2Ds + DBT, or 2Ds + DBT). The *p* values compare the detection rates between years.

			All Cancers		Invasive Cancers		DCIS		ADH	
Years	Tomo Type	Total	No	Yes	No	Yes	No	Yes	No	Yes
2012–2015	2DM + DBT	170	133	37	165	5	138	32	163	7
2015–2017	2DM + 2Ds + DBT	103	85	18	101	2	87	16	92	11
2017–2019	2Ds + DBT	131	95	36	124	7	102	29	121	10
Total		404	313	91	390	14	327	77	376	28
*p* =			0.191		0.392		0.448		0.096	

Notes: 2DM, 2D digital mammography; 2Ds, synthesized 2D mammography; DBT, digital breast tomosynthesis; DCIS, ductal carcinoma in situ; ADH, atypical ductal hyperplasia. Positive pathology results for each category are indicated by Yes. Negative pathology results for each category are indicated by No.

**Table 4:** Patient age characteristics by year and modality. Summary of patient characteristics by year and mammography modality for continuous characteristics. A: 2DM; B: 2Ds + DBT; C: 2DM + DBT; D: 2DM + 2Ds + DBT. For continuous parameters, for each year and modality, the table presents the number of patients (“N”), the minimum (“Min”) and maximum (“Max”) values, the quartiles (“q1” and “q3”), the median (“Med.”), the mean (“Mean”), and the standard deviation (“SD”), along with the number of missing values (“NA”), if any. For the continuous parameters, there is no evidence of a difference among years and modalities for age.

Variable	Year/Type	N	Min	q1	Med.	Mean	q3	Max	SD	#NA
Age	2012/A	215	30	47.000	54	56.237	65.000	89	11.820	0
	2012/C	184	35	47.000	54	54.859	61.250	85	10.143	0
	2015/A	155	39	47.500	55	56.284	64.000	84	10.523	0
	2015/D	115	37	48.000	59	58.217	67.000	82	10.842	0
	2017/A	89	40	49.000	55	55.124	61.000	81	8.488	0
	2017/B	181	32	49.000	56	57.282	65.000	82	10.488	0
*p* = 0.08	all	939	30	48.000	55	56.313	64.000	89	10.652	0

Modalities are represented as follows: A: 2DM; B: 2Ds + DBT; C: 2DM + DBT; D: 2DM + 2Ds + DBT.

**Table 5:** Patient characteristics (categorical) by year/type. Summary of patient characteristics by year and modality for categorical parameters. Patient characteristics were compared between study groups to evaluate whether patient characteristics differed between study populations.

Variable	Levels	2012/A (*n*)	2012/A (%)	2012/C (*n*)	2012/C (%)	2015/A (*n*)	2015/A (%)	2015/D (*n*)	2015/D (%)	2017/A (*n*)	2017/A (%)	2017/B (*n*)	2017/B (%)	All (*n*)	All (%)
Family History	No	37	17.3	47	25.5	25	16.1	27	23.5	13	14.6	34	18.8	183	19.5
	Yes	177	82.7	137	74.5	130	83.9	88	76.5	76	85.4	147	81.2	755	80.5
*p* = 0.13	all	214	100.0	184	100.0	155	100.0	115	100.0	89	100.0	181	100.0	938	100.0
Hormone Therapy	No	43	20.2	33	17.9	11	7.1	18	15.6	7	7.9	23	12.7	135	14.4
	Yes	170	79.8	151	82.1	143	92.9	97	84.3	82	92.1	158	87.3	801	85.6
*p* = 0.0025	all	213	100.0	184	100.0	154	100.0	115	100.0	89	100.0	181	100.0	936	100.0
Prior Mammogram	No	151	70.2	150	81.5	134	86.5	103	89.6	68	76.4	160	88.4	766	81.6
	Yes	64	29.8	34	18.5	21	13.6	12	10.4	21	23.6	21	11.6	173	18.4
*p* = 0.0005	all	215	100.0	184	100.0	155	100.0	115	100.0	89	100.0	181	100.0	939	100.0
Density	No	86	40.0	68	37.0	59	38.1	43	37.4	35	39.3	73	40.3	364	38.8
	Yes	129	60.0	116	63.0	96	61.9	72	62.6	54	60.7	108	59.7	575	61.2
*p* = 0.98	all	215	100.0	184	100.0	155	100.0	115	100.0	89	100.0	181	100.0	939	100.0

## Data Availability

Restrictions apply to the availability of these data. Data was obtained from [third party] and are available from the authors with the permission of third party.

## Key Finding

Four mammography modality combinations were assessed: 2DM, 2DM + DBT, 2DM + 2Ds + DBT, and 2Ds + DBT. Here, 2Ds technology appears to be non-inferior to 2DM in the identification of microcalcifications that underwent stereotactic biopsy. Direct implementation of 2Ds technology may be considered.

## Importance

This single-site, retrospective study analyzed the performance of four mammography modality combinations in identifying microcalcifications that underwent stereotactic biopsy.

## Abbreviations

2DM, 2D digital mammography; 2Ds, 2D synthesized mammography; DBT, 3D digital breast tomosynthesis.

## References

[1] Bernardi D., Macaskill P., Pellegrini M., Valentini M., Fantò C., Ostillio L., Tuttobene P., Luparia A., Houssami N. (2016). Breast cancer screening with tomosynthesis (3D mammography) with acquired or synthetic 2D mammography compared with 2D mammography alone (STORM-2): A population-based prospective study. Lancet Oncol..

[2] Bernardi D., Gentilini M.A., De Nisi M., Pellegrini M., Fantò C., Valentini M., Sabatino V., Luparia A., Houssami N. (2020). Effect of implementing digital breast tomosynthesis (DBT) instead of mammography on population screening outcomes including interval cancer rates: Results of the Trento DBT pilot evaluation. Breast.

[3] Gur D., Zuley M.L., Anello M.I., Rathfon G.Y., Chough D.M., Ganott M.A., Hakim C.M., Wallace L., Lu A., Bandos A.I. (2012). Dose Reduction in Digital Breast Tomosynthesis (DBT) Screening using Synthetically Reconstructed Projection Images: An Observer Performance Study. Acad. Radiol..

[4] Spangler M.L., Zuley M.L., Sumkin J.H., Abrams G., Ganott M.A., Hakim C., Perrin R., Chough D.M., Shah R., Gur D. (2011). Detection and Classification of Calcifications on Digital Breast Tomosynthesis and 2D Digital Mammography: A Comparison. Am. J. Roentgenol..

[5] Svahn T., Houssami N., Sechopoulos I., Mattsson S. (2015). Review of radiation dose estimates in digital breast tomosynthesis relative to those in two-view full-field digital mammography. Breast.

[6] Gennaro G., Bernardi D., Houssami N. (2018). Radiation dose with digital breast tomosynthesis compared to digital mammography: Per-view analysis. Eur. Radiol..

[7] Skaane P., Bandos A.I., Eben E.B., Jebsen I.N., Krager M., Haakenaasen U., Ekseth U., Izadi M., Hofvind S., Gullien R. (2014). Two-View Digital Breast Tomosynthesis Screening with Synthetically Reconstructed Projection Images: Comparison with Digital Breast Tomosynthesis with Full-Field Digital Mammographic Images. Radiology.

[8] Skaane P., Bandos A.I., Niklason L.T., Sebuødegård S., Østerås B.H., Gullien R., Gur D., Hofvind S. (2019). Digital Mammography versus Digital Mammography Plus Tomosynthesis in Breast Cancer Screening: The Oslo Tomosynthesis Screening Trial. Radiology.

[9] Zuckerman S.P., Conant E.F., Keller B.M., Maidment A.D.A., Barufaldi B., Weinstein S.P., Synnestvedt M., McDonald E.S. (2016). Implementation of Synthesized Two-dimensional Mammography in a Population-based Digital Breast Tomosynthesis Screening Program. Radiology.

[10] Abdullah P., Alabousi M., Ramadan S., Zawawi I., Zawawi M., Bhogadi Y., Freitas V., Patlas M.N., Alabousi A. (2021). Synthetic 2D Mammography Versus Standard 2D Digital Mammography: A Diagnostic Test Accuracy Systematic Review and Meta-Analysis. Am. J. Roentgenol..

[11] Zuley M.L., Guo B., Catullo V.J., Chough D.M., Kelly A.E., Lu A.H., Rathfon G.Y., Spangler M.L., Sumkin J.H., Wallace L.P. (2014). Comparison of Two-dimensional Synthesized Mammograms versus Original Digital Mammograms Alone and in Combination with Tomosynthesis Images. Radiology.

[12] Peters S., Hellmich M., Stork A., Kemper J., Grinstein O., Püsken M., Stahlhut L., Kinner S., Maintz D., Krug K.B. (2017). Comparison of the Detection Rate of Simulated Microcalcifications in Full-Field Digital Mammography, Digital Breast Tomosynthesis, and Synthetically Reconstructed 2-Dimensional Images Performed With 2 Different Digital X-ray Mammography Systems. Investig. Radiol..

[13] Lai Y.-C., Ray K.M., Lee A.Y., Hayward J.H., Freimanis R.I., Lobach I.V., Joe B.N. (2018). Microcalcifications Detected at Screening Mammography: Synthetic Mammography and Digital Breast Tomosynthesis versus Digital Mammography. Radiology.

[14] Dodelzon K., Simon K., Dou E., Levy A.D., Michaels A.Y., Askin G., Katzen J.T. (2020). Performance of 2D Synthetic Mammography versus Digital Mammography in the Detection of Microcalcifications at Screening. Am. J. Roentgenol..

[15] Vedantham S., Karellas A., Vijayaraghavan G.R., Kopans D.B. (2015). Digital Breast Tomosynthesis: State of the Art. Radiology.

[16] Freer P.E., Riegert J., Eisenmenger L., Ose D., Winkler N., Stein M.A., Stoddard G.J., Hess R. (2017). Clinical implementation of synthesized mammography with digital breast tomosynthesis in a routine clinical practice. Breast Cancer Res. Treat..

[17] Glynn C.G., Farria D.M., Monsees B.S., Salcman J.T., Wiele K.N., Hildebolt C.F. (2011). Effect of Transition to Digital Mammography on Clinical Outcomes. Radiology.

